# OX40 ligand expression abrogates the immunosuppressive function of retinal pigment epithelium

**DOI:** 10.1186/1869-5760-3-12

**Published:** 2013-01-15

**Authors:** Matthew A Cunningham, Zhuqing Li, Baoying Liu, Steven Yeh, Robert B Nussenblatt

**Affiliations:** 1 Vitreoretinal Service, Department of Ophthalmology and Visual Sciences, The University of Iowa Hospitals & Clinics, Iowa City, IA, 52242, USA; 2 National Institute of Allergy and Infectious Diseases, National Institutes of Health, Bethesda, MD, 20892, USA; 3 Laboratory of Immunology, National Eye Institute, National Institutes of Health, 10 Center Dr. Bldg 10, 10D45, Bethesda, MD, 20892, USA; 4 Emory Eye Center, Section of Vitreoretinal Surgery and Disease, Atlanta, GA, 30322, USA

**Keywords:** OX40 ligand, Uveitis, Retinal pigment epithelium, Tumor necrosis factor ligand, *in vitro*

## Abstract

**Background:**

This study aims to investigate the role of OX40 ligand (OX40L) in ocular inflammation via abrogation of retinal pigment epithelium (RPE)-mediated immunosuppression using an *in vitro* expression approach. OX40L cDNA was polymerase chain reaction-amplified and cloned into an eYFP fusion vector. Cultured retinal pigment epithelial cells (ARPE-19) were transfected with the vector. Total RNA from unstimulated or inflammatory cytokine-stimulated ARPE cells were isolated and analyzed for OX40L expression by reverse transcription-polymerase chain reaction. Peripheral blood mononuclear cells (PBMCs) were isolated from healthy human donors. Human ARPE cells (±OX40L ± GITR ligand (GITRL) expression) and PBMCs were co-cultured for *in vitro* proliferation studies.

**Results:**

Polymerase chain reaction confirmed the insertion of the OX40L gene into the fusion vector. Flow cytometry and fluorescence microscopy further confirmed surface expression of OX40L on ARPE cells after transfection. OX40L expression was induced in the RPE cells stimulated with pro-inflammatory cytokines. In the co-culture studies, there was a significant reversal (20% to 30%) of the RPE-induced suppression of activated PBMCs when the ARPE cells were transfected with OX40L. When both OX40L and GITRL were concomitantly transfected into ARPE cells, there was an additive reversal of RPE-mediated T cell suppression, when compared to the reversal caused by RPE cells expressing either OX40L alone or GITRL alone.

**Conclusions:**

Using an *in vitro* approach, we found that OX40L causes an abrogation of the RPE-mediated immunosuppression. OX40L appears to be regulated in the ARPE-19 cell line and may play an important role in the pathogenesis of various ocular inflammatory conditions.

## Background

OX40 ligand (OX40L) is a member within the TNF ligand superfamily [1-4] and plays an important role in the induction of co-stimulatory signaling during T cell and antigen-presenting cell (APC) interactions [5-9]. Prior studies have demonstrated that OX40L is expressed predominantly on CD4+ T cells [10,11]. In addition to its role in co-stimulatory signaling, OX40/OX40L interaction is closely involved in effector functions [12-15]. OX40L expression has been observed on APCs including dendritic cells [16,17], B cells [12], and microglial cells [18], but has also been found on non-lymphoid cells (i.e., endothelial cells) [19].

Recently, its potential role in the pathogenesis of immune-mediated ophthalmic diseases including uveitis, experimental allergic conjunctivitis, and corneal graft rejection has been investigated [20-23]. A recent study by Zhang et al. described the activation of ovalbumin-specific T cells with OX40-activating antibody and found that OX40-stimulated lymphocytes elicited more ocular inflammation compared to donor cells without OX40 activation. Moreover, flow cytometry showed that Th17 cells expressed OX40 ligand, and interleukin-17 neutralizing antibody was successful in the attenuation of OX40-mediated uveitis [22]. In another study of experimental autoimmune uveitis, OX40 activation was found to promote the expansion uveitogenic memory T cells, as evidenced by the upregulation of IL-7R in CD4^+^CD44^+^ lymphocytes [23].

Other animal models of ocular inflammation have been described. In a murine model of experimental allergic conjunctivitis, *in vivo* stimulation of OX40 with an anti-OX40 antibody led to the exacerbation of ocular surface inflammatory disease, while antibody blockade of the OX40/OX40L interaction inhibited disease expression [20]. Hattori et al. characterized OX40/OX40L interaction in a murine model of corneal transplant rejection. They describe significantly reduced corneal allograft rejection in mice treated with a monoclonal antibody targeting murine OX40L when compared to control mice. In addition, OX40 ligand-deficient mice demonstrated a decreased risk of allograft rejection when transplanted with wild-type donor corneas. An *in vitro* study also revealed that treatment with anti-OX40L monoclonal antibody reduced the proliferative response and interferon-gamma production of lymph node cells after donor alloantigen stimulation. These studies were suggestive that OX40L blockade prolonged graft survival via inhibition of recipient T cell activation [21].

Thus, while OX40L and OX40 in activating inflammation has been suggested by several animal models of systemic and ophthalmic inflammation, the precise pathways by which OX40L leads to these ocular inflammatory sequelae warrants further investigation. The eye is an immune-privileged organ, and multiple mechanisms are thought to contribute to this privilege [24-26] including retinal pigment epithelium (RPE)-mediated suppression of T cell activation [27], induction of apoptosis of activated T cells via Fas-FasL interactions [28], and inhibition of T cell proliferation by a cell-contact-dependent mechanism [29]. Using an *in vitro* expression system previously described in a report for GITR ligand [30], another important member of the TNF superfamily, we sought to further investigate the role of OX40L and its role in ocular inflammation, specifically in its ability to abrogate RPE-mediated immunosuppression. We found that OX40L causes abrogation of RPE-mediated immunosuppression and appears to be regulated within RPE cells.

## Results

### Confirmation of OX40L insertion into eYFP vector

**Figure 1:**
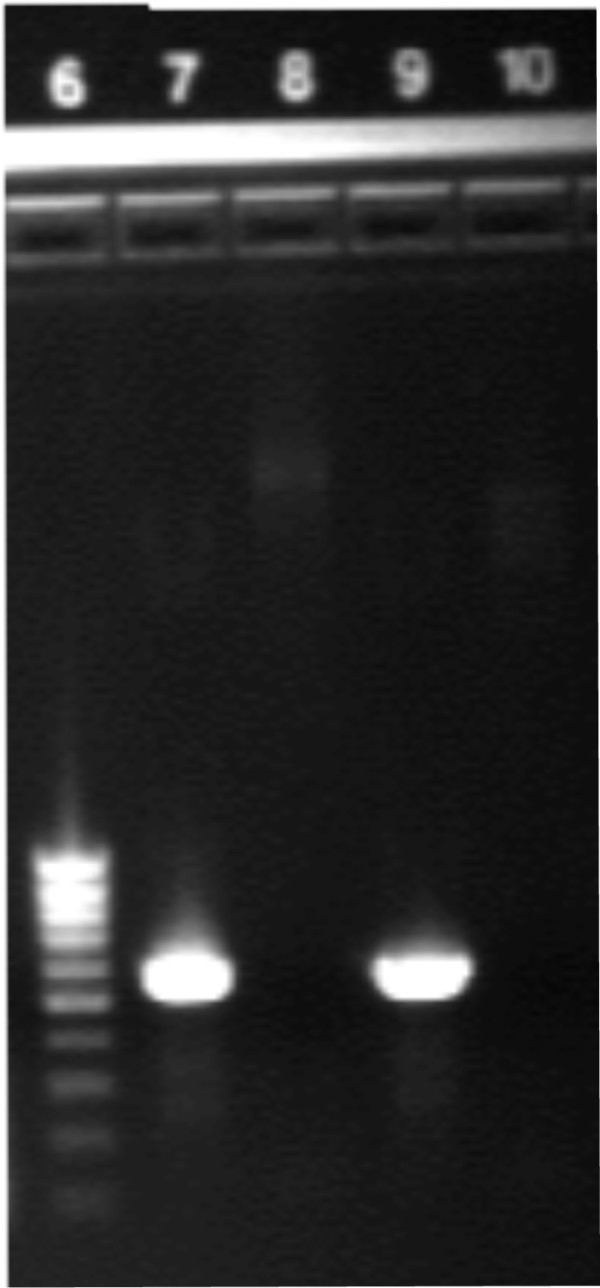
**OX40L gene identification by PCR.** PCR performed on purified plasmid with and without OX40L insert. Note the presence of OX40L in lanes 7 and 9. (Lane 6, low range DNA ladder; lane 7, eYFP-C1 + OX40L insert; lane 8, eYFP-C1 plasmid; lane 9, eYFP-N1 +OX40L insert; lane 10, eYFP-N1 plasmid).

To confirm the correct insertion of the OX40L gene into the eYFP vector, polymerase chain reaction (PCR) was performed on the purified products. OX40L was detected using PCR (Figure 1), along with restriction enzyme (BamH1 and HindIII) overnight digestion. The gene was identified in both the eYFP-C1 and eYFP-N1 vectors.

### Expression of OX40L on ARPE-19 cells

Using flow cytometry and a biotinylated anti-OX40L antibody, we found that the expression of OX40L was undetectable (data not shown). After transfection, OX40L was expressed on RPE cells, and the expression was monitored using both flow cytometry and fluorescence microscopy.

**Figure 2:**
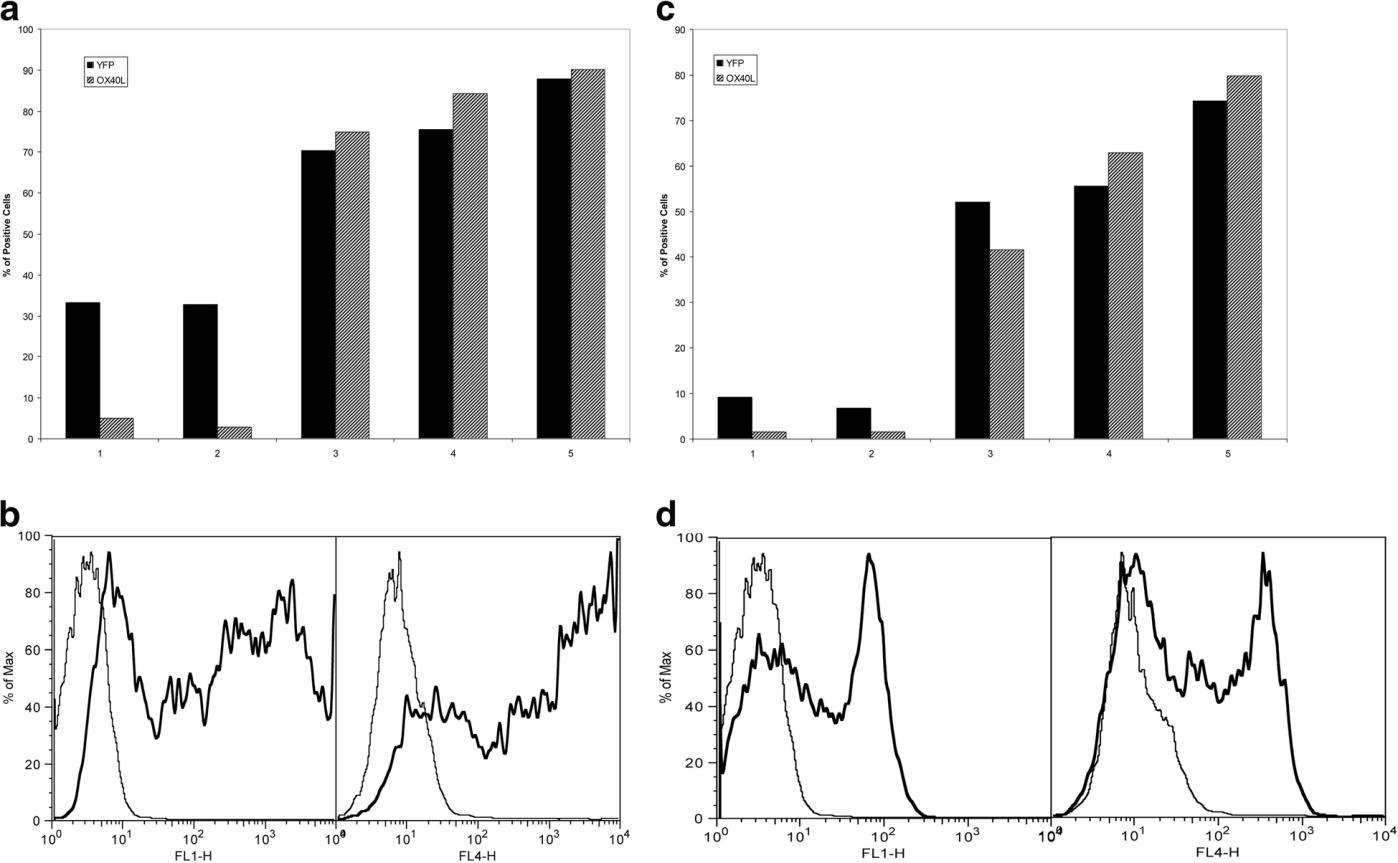
**Time trial after vector transfection. (a)** Bar graph representing the expression pattern of the fluorescent protein (YFP) and OX40L after transfection of eYFP-C1-OX40L into RPE at five time points (time point 1 = 30 min, time point 2 = 1 h, time point 3 = 4 h, time point 4 = 6 h, and time point 5 = 24 h). Time-dependent increased expression is observed. **(b)** Flow cytometric analysis at 4 h after transfection of RPE cells with eYFP-C1-OX40L, with YFP expression (bold) in the left panel and OX40L expression (bold) in the right panel (the thinner lines represent RPE that was not transfected). **(c)** Bar graph expression pattern of YFP and OX40L after transfection of eYFP-N1-OX40L into RPE cells at the same time points. **(d)** Flow cytometric analysis at 4 h after transfection of RPE cells with eYFP-N1-OX40L.

In a time and dosage trial, after verified transfection of the eYFP-OX40L construct into RPE cells, surface expression of OX40L was demonstrated as early as 4 h after transfection (see Figure 2). The expression of our protein was primarily cytosolic prior to the 4-h mark. This was demonstrated by the flow cytometric detection of the eYFP fluorescent protein as early as 30 min after transfection. During the dosage trial, transfecting a dose of 0.5 μg per 0.6 × 10^6^ RPE cells led to an expression >90% (data not shown). Larger transfection dosages led to a plateau effect, and dosages >5 μg, in the eYFP-OX40L-C1 construct, had a toxic effect to the RPE cells (data not shown).

### Cytokine-mediated regulation of OX40L RNA in cultured RPE

**Figure 3:**
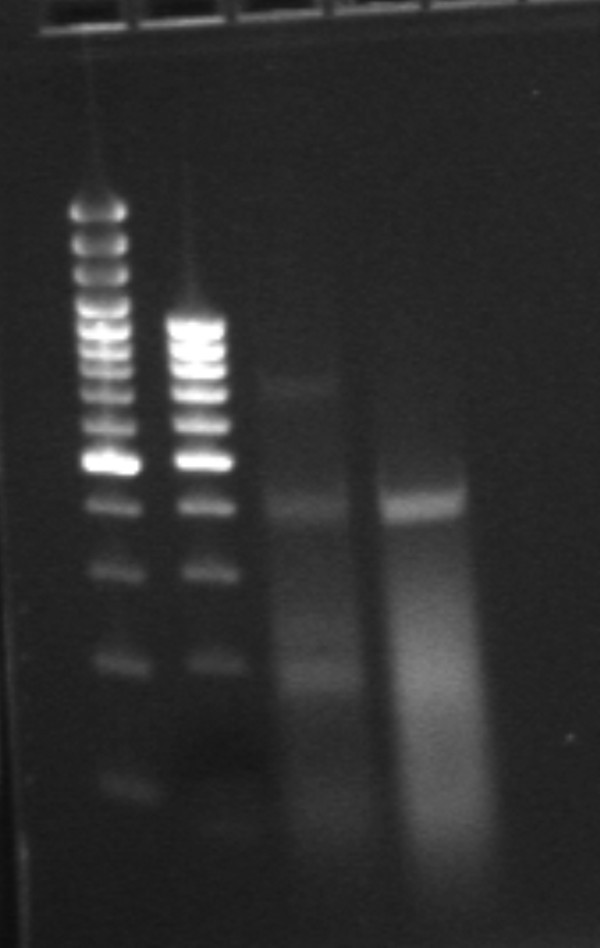
**RT-PCR demonstrated expression of OX40L in the RNA of ARPE cells stimulated by pro-inflammatory cytokines.** Lanes 1 and 2 are two different DNA ladders; lanes 3 and 4 represent two different concentrations of the same RNA from stimulated RPE cells.

Using reverse transcription-polymerase chain reaction (RT-PCR), the OX40L RNA content in resting, unstimulated RPE cells and in RPE cells stimulated for 24 h with high levels of the pro-inflammatory cytokines IL-1α, TNF-α, and IFN-γ were compared. Constitutive expression of OX40L RNA was not observed in unstimulated RPE cells; however, in the stimulated RPE cells, OX40L was expressed (Figure 3).

### Transient expression of OX40L in RPE cell reverse RPE-induced suppression of PBMCs

**Figure 4:**
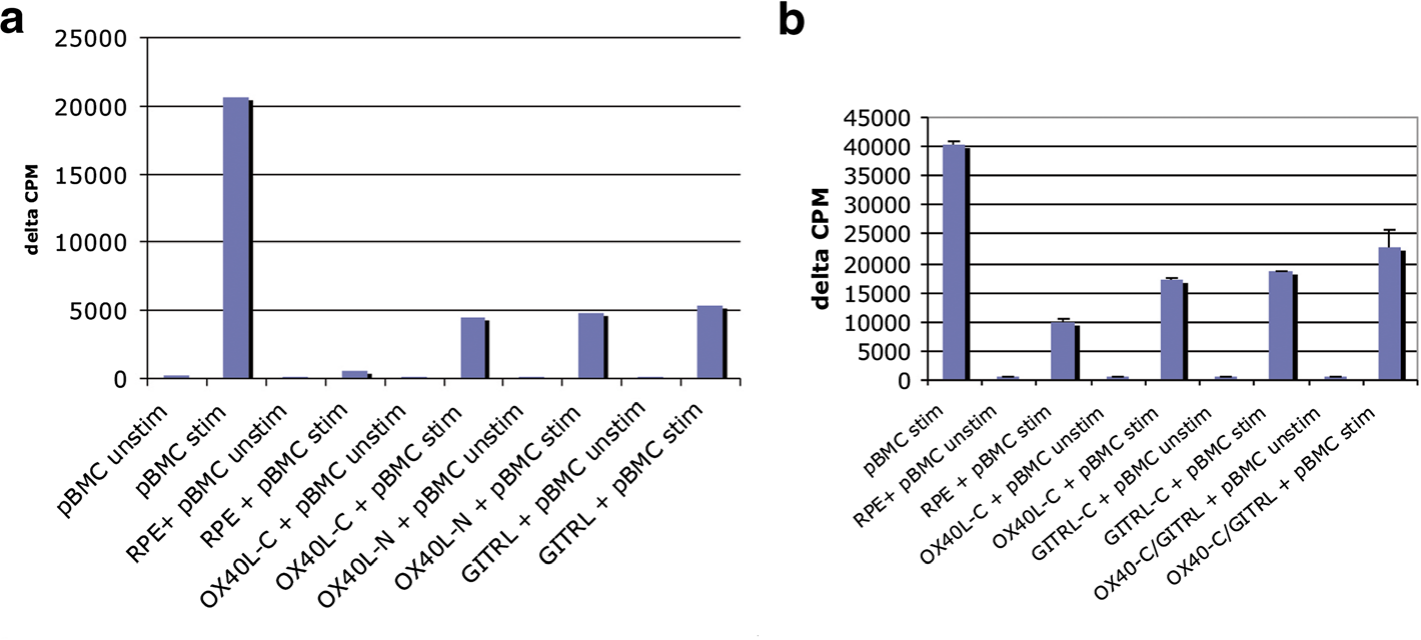
**ARPE-19 and PBMC co-culture study results. (a)** OX40L-C + PBMC abrogates the immunosuppressive effect of RPE alone. No difference was observed between OX40L (C- or N- vectors) or GITR ligand. An approximately 20% to 30% reversal of RPE-mediated immunosuppression is seen when stimulated pBMCs were co-cultured with RPE-OX40L vector. **(b)**A repeat experiment shows that concomitant transfection of both eYFP-C1-OX40L and eYFP-C1-GITRL vectors into RPE cells resulted into an additive reversal of immunosuppression of stimulated pBMCs, when comparing it to either RPE transfected with OX40L-C or GITRL alone. (pBMC unstim, unstimulated peripheral blood mononuclear cells; pBMC stim, stimulated pBMC with anti-CD3 and anti-CD28; RPE, retinal pigment epithelial cells without transfection; OX40L-C, RPE cells transfected with eYFP-C1-OX40L; OX40L-N, RPE cells transfected with eYFP-N1-OX40L; GITRL, RPE cells transfected with eYFP-C1-GITRL).

A co-culture of RPE cells (with or without OX40L expression) and PBMCs (with or without anti-CD3/anti-CD28 stimulation) was performed. RPE cells without OX40L expression, when co-cultured with either stimulated or unstimulated PBMCs, demonstrated the greatest suppressive activity (Figure 4). When RPE cells were transfected with either the eYFP-C1-OX40L or the eYFP-N1-OX40L construct, an approximately 20% to 30% reversal of the RPE-mediated suppression of PBMCs was observed. A significant difference in the abrogation of RPE-mediated suppression was not observed between the RPE transfected with the eYFP-N1-OX40L and the eYFP-C1-OX40L vectors. Interestingly, when both OX40L-C and GITR ligand (GITRL) were transfected into RPE cells, an increase in the reversal of RPE-mediated suppression was observed when compared to the reversal demonstrated with RPE transfected with OX40L-C or GITRL alone (data not shown).

## Discussion

A significant body of evidence has supported the role of RPE cells in the maintenance of ocular immune privilege [27,31-33]. RPE cells are capable of acting as efficient APCs expressing MHC class II molecules, and exposure to varying cytokine milieus may lead to RPE-mediated T cell activation or T cell regulation [31,34].

A number of the TNF receptor family members are thought to promote inflammatory processes via T cell co-stimulation, proliferation, and differentiation. The TNF-R/TNF-α interaction has been well described both in experimental animal models and patients with ocular inflammatory diseases. We previously showed that another member of the TNF receptor family, GITRL, may also play a role in ocular inflammation. Specifically, GITRL expression was able to abrogate the immunosuppressive function of RPE cells on CD3+ T cells via decreased TGF-beta expression in RPE/CD3+ T cell co-cultures and increased production of pro-inflammatory cytokines and chemokines [30]. In patients with uveitis, GITR was found to be an activation marker for CD4+ T cells and was co-expressed with CD25 on CD4+ T cells in peripheral blood. The presence of GITR was also found to be correlated with disease course in uveitis [35].

In this study, we investigated whether OX40L, another member of the TNF family involved with inflammatory disease states, may play a role in the pathogenesis of ocular inflammation. However, current human RPE cell lines do not express OX40L at the protein level. Moreover, in unactivated human RPE cells, we did not observe OX40L at the RNA level. Nonetheless, after exposure of RPE cells to a pro-inflammatory cytokine milieu (IL-1α,TNF-α, and IFN-γ), we did observe OX40L RNA expression. This represents the first demonstration of OX40L RNA in ocular tissue and suggests that OX40L expression is an inducible phenomenon in the setting of localized inflammation.

To investigate the functional role of OX40L in RPE cells, we used an *in vitro* expression approach to mimic a pro-inflammatory environment. The use of a fluorescent-tagged fusion vector allowed us to confirm the presence of OX40L transfection into RPE cells and its expression using flow cytometry. In RPE-PBMC co-culture experiments, we found that RPE cells expressing OX40L (after transfection) reversed the immunosuppression afforded by RPE cells that did not express OX40L. PBMCs rather than purified T cells were used to simulate an extracellular milieu that would more closely mimic the ocular immune environment *in vivo*. OX40L was transfected in ARPE cells, which normally do not express OX40L, to mimic the ocular inflammatory scenario.

Recent reports have shown that the OX40-OX40L interaction may play a role in a number of ocular inflammatory processes including corneal transplant rejection [21], experimental allergic conjunctivitis [20], herpes stromal keratitis [36], and uveitis. Several recent studies have described the relationship of OX40 ligand in the exacerbation and prolongation of animal models of uveitis as well as its role in augmenting the pro-inflammatory Th17 cytokine response.

Moreover, several other mechanisms by which OX40L perpetuates the inflammatory process in other biologic systems have been suggested. OX40L was recently found to inhibit the generation of IL-10 producing CD4+ type 1 regulatory T cells from naive T cells. In addition, OX40L inhibited IL-10 production in differentiated IL-10 producing regulatory T cells [37]. Other proposed functions of OX40L include its role in the T cell effector response, T cell-APC co-stimulatory signaling, the T cell-dependent humoral responses [38], and the promotion of CD4+ T cell longevity [39].

## Conclusions

The numerous mechanisms by which OX40L may activate inflammation or abrogate immunosuppression, particularly in the complex ocular immune environment in which immune privilege is critical to immunoregulation, remain incompletely understood. We have demonstrated that a pro-inflammatory cytokine milieu is sufficient to induce RNA expression of OX40L by RPE cells, and its expression appears to be highly regulated. In addition, our co-culture experiments with activated PBMCs and transfected RPE cells demonstrate that OX40L expression is associated with dampening of RPE-mediated immunosuppression, a novel finding that warrants additional investigation. This study, along with prior work on the role of GITRL in ocular inflammation, adds to the growing evidence that other members of the TNF ligand superfamily besides TNF-α may play a role in ocular immunity. Further studies will be needed to determine the precise role of OX40L in human uveitis and whether modulation of OX40L pathways may have therapeutic implications.

## Methods

### Reagents, cell lines, and antibodies

The retinal pigment epithelial cells (ARPE-19 cells) were originally obtained from ATCC (Manassas, VA, USA) and were cultured in minimum essential medium (MEM) with 10% fetal bovine serum (FBS; Gemini Bio-products, West Sacramento, CA, USA), 1X nonessential amino acids, 1X antibiotics, and 2 mM glutamine (Biosource International, Bethesda, MD, USA) at 37°C in 5% CO_2_. Biotinylated polyclonal goat anti-human OX40L antibody (Catalog # BAF1054) was obtained from R & D systems (Minneapolis, MN, USA). IL-1α, TNF-α, and IFN-γ were purchased from Biosource International (Camarillo, CA, USA). The media and FBS were purchased as lipopolysaccharide-free-grade reagents.

### Plasmid

The fusion vectors eYFP-C1 and eYFP-N1 (BD Biosciences Clontech, San Jose, CA, USA) were used so that we could readily assess the presence of stably inserted OX40L DNA. The eYFP-C1 vector attaches the tag protein eYFP to the C-terminal (intracellular) region of the inserted gene, while the eYFP-N1 vector attaches eYFP protein to the N-terminal (extracellular) region of the inserted gene.

### Cloning of full-length hOX40L cDNA

Full-length OX40L was amplified from a human HTLV-1-infected T cell lymphoma (MT-2) cell line [40] using hOX40L specific primers:

hOX40L to insert into the eYFP-C1 vector

5′-CCCAAGCTTCCATGGAAAGGGTCCAACCCC-3′ (forward) and

5′-CGCGGATCCTCAAAGGACACAGAATTCACC-3′ (reverse).

hOX40L to insert into eYFP-N1 vector

5′-CCCAAGCTTGCCACCATGGAAAGGGTCCAACCCC-3′ (forward) and

5′-CGCGGATCCGCAAGGACACAGAATTCACCAGG-3′ (reverse).

RT-PCR was performed with the one-step RT-PCR kit (Promega, Madison, WI, USA) according to the manufacturer's instructions. The amplification procedure for RT-PCR was as follows: 48°C for 75 min, 94°C for 2 min and 30 s followed by cycles of the following: 94°C for 30 s, 50°C for 45 s, and 72°C for 1 min. There was a total of 40 cycles.

Total human OX40L product (552 bp) was purified using the QIAquick PCR purification kit (Qiagen Inc., Santa Clarita, CA, USA), and full-length OX40L was subcloned in frame into either the eYFP-C1 or eYFP-N1 vector. With eYFP-C1, the gene was inserted between the eYFP coding sequence and the stop codon, and with eYFP-N1, the OX40L gene was inserted between the immediate early promoter of CMV and the eYFP coding sequence.

### Transfection of ARPE-19 cell line

To facilitate the expression of hOX40L in RPE cells, human adult retinal pigment epithelial cell line ARPE-19 was transfected with the construct eYFP-C1-OX40L or with eYFP-N1-OX40L using the Nucleofector transfection system (Amaxa Inc, Gaithersburg, MD, USA). Prior to transfection, RPE cells were grown to 80% to 85% confluence and resuspended in 2 to 3× 10^6^ cells/0.1 mL Nucleofector Solution V with 2 μg of eYFP-OX40L construct. The solution was transferred to a sterile electroporation cuvette and transfected using program #T20 according to manufacturers’ instructions. The transfected cells were then plated in the culture medium (MEM, 10% FBS, 1X antibiotics) on six-well culture plates.

### Expression of OX40L

Flow cytometry was utilized to analyze the surface expression of OX40L in RPE cells and to study the OX40L expression patterns in a time trial and at varying transfection dosages. For the time trial, RPE cells (2 × 10^6^ cells) transfected with either eYFP-C1-OX40L or eYFP-N1-OX40L for different time points (30 min, 1, 4, 6, and 24 hr) were incubated in cold PBS (pH 7.4) with 0.5% BSA with or without biotinylated anti-OX40L antibody for the detection of surface OX40L. Cells were washed and fixed with 1% paraformaldehyde, counted via the FACS Calibur flow cytometer (Becton Dickinson, San Jose, CA, USA), and analyzed using FlowJo software (TreeStar, San Jose, CA, USA). Total RNA from cultured RPE cell lines was isolated with an RNA isolation kit (RNeasy, Qiagen Inc.) using the manufacturers’ instructions. Prior to RNA isolation, one cell line was stimulated for 24 h with 5.0 ng/mL of IL-1α,5.0 ng/mL of TNF-α, and 25 ng/mL of IFN-γ. Another cultured cell line was transfected with 1 μg eYFP-OX40L construct and served as the positive control for the RT-PCR. The last cultured cell line was not treated with pro-inflammatory cytokines and represented resting RPE cells. Using 1 μg of RNA, initially, RT-PCR was used to screen for the product (OX40L). The amplification procedure was as follows: 48°C for 75 min, 94°C for 2 min and 30 s followed by cycles of the following: 94°C for 30 s, 50°C for 45 s, and 72°C for 1 min. There was a total of 40 cycles. The primers used for amplification were from different exons and their sequences were as follows: OX40L, 5′-TCACCTACATCTGCCTGCACTTCTCTGCTCTT-3′ and 5′-ATCAGTTCTCCGCCATTCACA-3′.

### Isolation of peripheral blood mononuclear cells

Human peripheral blood mononuclear cells (PBMCs) were isolated from buffy coat obtained from normal, healthy donors (NIH blood bank) using the Ficoll gradient centrifugation.

### RPE-PBMC co-culture studies

An RPE-PBMC co-culture system was used for the *in vitro* proliferation studies. ARPE-19 cells were grown to 80% to 85% confluence and transfected with eYFP-OX40L vector. Twelve hours after transfection, the RPE cells were lethally irradiated (9,000 rads), washed with FBS-supplemented RPMI 1640 medium and plated in 96-well flat-bottomed culture plates, using enough RPE cells to obtain complete confluence. Isolated PBMCs (2 ×10^5^cells/well) were co-cultured with the already plated RPE cells. To a set number of cultures, anti-CD28 antibody (2 μg/mL) and anti-CD3 antibody (2 μg/mL) were added. After 3 days of culturing at 37°C in 5% CO_2_, the cultures were pulsed with ^3^H thymidine (Amersham, Buckinghamshire, UK) and then cultured for another 8 to 12 h. The cells were harvested, and uptake of ^3^H thymidine was measured by a beta counter (Perkin Elmer Life Sciences, Waltham, MA, USA). The effect of RPE cells (± transfected with OX40L) on PBMCs (± stimulation) was expressed as delta counts per minute (Δcpm). All experiments were performed in triplicate and summarized herein.

All research conformed to the ARVO statement on human and animal research and the Declaration of Helsinki. Samples from normal donors were obtained from the National Institutes of Health blood bank after informed consent was obtained.

## Competing interests

The authors declare that they have no competing interests.

## Authors’ contributions

MC participated in the study design and coordination, carried out the experiments, and drafted the manuscript. ZL conceived of the study and participated in the study design, various experiment portions, and in the revision of the manuscript as well as the statistical analysis. BL participated in the experiments and in the revision of the manuscript. SY participated in the various experiments, in intellectual discussion, and in the drafting/revision of the manuscript. RN participated in the design of the study, in intellectual discussion, and held a supervisory role. All authors read and approved the final manuscript.
